# Sesamol Increases Ucp1 Expression in White Adipose Tissues and Stimulates Energy Expenditure in High-Fat Diet-Fed Obese Mice

**DOI:** 10.3390/nu12051459

**Published:** 2020-05-18

**Authors:** Dong Ho Lee, Seo-Hyuk Chang, Dong Kwon Yang, No-Joon Song, Ui Jeong Yun, Kye Won Park

**Affiliations:** 1 Department of Food Science and Biotechnology, Food Clinical Research Center, Sungkyunkwan University, Suwon 16419, Korea; zestcoren@gmail.com (D.H.L.); adc153@naver.com (S.-H.C.); songnj413@naver.com (N.-J.S.); yunc@skku.edu (U.J.Y.); 2 Department of Veterinary Pharmacology and Toxicology, College of Veterinary Medicine, Chonbuk National University, Iksan, Jeollabuk-do 54596, Korea; dkyang0502@gmail.com

**Keywords:** sesamol, uncoupling protein 1, Nrf2, energy expenditure, obesity, metabolic diseases

## Abstract

Sesamol found in sesame oil has been shown to ameliorate obesity by regulating lipid metabolism. However, its effects on energy expenditure and the underlying molecular mechanism have not been clearly elucidated. In this study, we show that sesamol increased the uncoupling protein 1 (*Ucp1*) expression in adipocytes. The administration of sesamol in high-fat diet (HFD)-fed mice prevented weight gain and improved metabolic derangements. The three-week sesamol treatment of HFD-fed mice, when the body weights were not different between the sesamol and control groups, increased energy expenditure, suggesting that an induced energy expenditure is a primary contributing factor for sesamol’s anti-obese effects. Consistently, sesamol induced the expression of energy-dissipating thermogenic genes, including *Ucp1*, in white adipose tissues. The microarray analysis showed that sesamol dramatically increased the Nrf2 target genes such as *Hmox1* and *Atf3* in adipocytes. Moreover, 76% (60/79 genes) of the sesamol-induced genes were also regulated by tert-butylhydroquinone (tBHQ), a known Nrf2 activator. We further verified that sesamol directly activated the Nrf2-mediated transcription. In addition, the *Hmox1* and *Ucp1* induction by sesamol was compromised in Nrf2-deleted cells, indicating the necessity of Nrf2 in the sesamol-mediated *Ucp1* induction. Together, these findings demonstrate the effects of sesamol in inducing *Ucp1* and in increasing energy expenditure, further highlighting the use of the Nrf2 activation in stimulating thermogenic adipocytes and in increasing energy expenditure in obesity and its related metabolic diseases.

## 1. Introduction

An increased energy intake combined with less energy expenditure leads to weight gain and obesity [1,2,3,4]. Less energy input or enhanced energy expenditure can prevent obesity and improve glucose metabolism [3,5,6]. Exercise and diets are the most effective way to prevent weight gain but such life style changes are often difficult to make [1]. Most currently available approaches for weight management target the brain for appetite control or the intestine for absorption reduction, but are associated with adverse effects such as headache, depression, nausea, and diarrhea [7,8,9]. Thus, increased metabolic rates preferentially beyond the nervous system and intestine that also contribute to an enhanced energy expenditure are being considered as an alternative [10].

Adipose tissue is composed of metabolically active thermogenic adipocytes and less active white adipocytes [11,12,13]. Thermogenic adipocytes, namely brown and beige adipocytes, dissipate heat by uncoupling the electron transport and ATP synthesis. The uncoupling protein 1 (Ucp1)-localized inner mitochondrial membrane of brown or beige adipocytes decreases the proton gradient and induces fast fatty acid oxidation, leading to heat dissipation [14]. Ucp1-deficient mice are extremely sensitive to acute cold exposure, indicating the role of Ucp1 in thermogenesis [15,16]. Conversely, adipose-selective Ucp1 transgenic mice exhibit less fat mass and an increased energy expenditure [17]. Accordingly, Ucp1 induction is thought to induce energy expenditure and may be useful in strategies to prevent obesity and its related metabolic diseases [11,14].

Recent studies show that phytochemicals can stimulate the induction of beige adipocytes or the activation of brown adipocytes [11]. For example, plant-derived phytochemicals, including berberine, butein, capsaicin, fucoxanthin, and oxyresveratrol, or endogenously found small molecules such as serotonin, lactate, and adenosine, have been identified for their potential to activate/induce Ucp1 in brown or white adipocytes [18]. Although the effectiveness of these molecules is yet to be proven in humans, these phytochemicals can potentially be used in therapeutic applications in humans. 

Nuclear factor erythroid-derived 2-like 2 (Nrf2) is a transcription regulator that drives the transcription of antioxidant-related genes [19,20]. Nrf2, under normal conditions, is kept in the cytoplasm by Kelch-like ECH-associated protein 1 (Keap1) and degraded by the ubiquitinase Cullin3. Under oxidative stress, Nrf2 is released from the Keap1–Cullin3 complex and accumulated, followed by a translocation into the nucleus. Nrf2 binds to the antioxidant response element (ARE) of the upstream region of the target genes to drive their transcription. The activation of Nrf2 induces the expression of cytoprotective antioxidation-related genes such as the glutamate-cysteine ligase modifier subunit (Gclm), sulfiredoxin 1 (Srxn1), superoxide dismutases, catalase, glutathione reductase, NAD(P)H quinone oxidoreductase 1 (Nqo1), and heme oxygenase-1 (Hmox1) [20]. 

Sesamol is a natural polyphenolic compound in sesame seeds and sesame oil. It has been shown to exhibit anti-oxidative, anti-cancer, anti-mutagenic, and anti-fungal activities [21]. Sesamol inhibits adipogenic differentiation in 3T3-L1 cells by regulating ERK 1/2, JNK, p38, and AMPK activities [22]. Sesamol in the drinking water of the high-fat diet (HFD)-fed C57BL6/J mice mitigates the body weight gain and insulin resistance by altering the mitochondria-related genes’ expressions [23,24]. Sesamol attenuates diet-induced cardiometabolic syndrome in rats by modulating the lipogenic- and inflammatory-related genes [25]. However, the exact mechanism underlying the anti-obese effects and energy expenditure of sesamol has not been clearly elucidated. 

In this study, to elucidate the molecular mechanisms of anti-obese effects of sesamol, we assessed the effects of sesamol on the *Ucp1* expression in white adipose tissues and energy expenditure in mice. Our data demonstrated that the oral administration of sesamol for 3 weeks, when body weight differences are not evident, still increased energy expenditure, indicating that the higher energy expenditure is the primary cause for the prevention of obesity and its associated metabolic dysregulation in HFD-fed obese mice. In the molecular mechanism studies, we further presented that sesamol activated Nrf2 to induce thermogenic genes in adipocytes. Together, our results show that sesamol increases energy expenditure by acting on Nrf2 and further support the potential use of sesamol and Nrf2 activators in treating obesity and metabolic diseases.

## 2. Materials and Methods 

### 2.1. Cell Culture and Adipocyte Differentiation

C3H10T1/2 cells, T37i brown adipocytes, and primary adipocytes were maintained as previously described [26,27]. Nrf2 wild type and Nrf2 knockout (KO) mouse embryonic fibroblasts (MEFs) were described. C3H10T1/2 cells were induced into adipocytes in Dulbecco’s modified Eagle’s medium (DMEM) (Hyclone, Logan, UT, USA) media supplemented with 10% FBS, 1 μM dexamethasone (Sigma, St. Louis, MO, USA), 0.5 mM isobutyl-1-methylxanthine (Sigma), 5 μg/mL insulin (Sigma), and 20nM GW1929 (Sigma). After 48 h, the differentiating cells were refreshed with media containing DMEM, 10% FBS, 5 μg/mL insulin, and 20 nM GW1929. T37i cells were maintained in DMEM/F-12, 10% CS, and 1% penicillin/streptomycin. Confluent cells were induced into brown adipocytes in DMEM/F-12, 10% CS, 5 μg/mL insulin, and 2.5 nM T3. After adipocyte differentiation for 6 to 8 days, cells were treated with sesamol or tert-butylhydroquinone (tBHQ) (Sigma). MTT assay using 3-(4,5-dimethylthiazol-2-yl)-2,5-diphenyltetrazolium-bromide (MTT, Sigma) and mitochondrial staining using CytoPainter (ab112145; Abcam, Cambridge, UK) were performed as previously described [28].

### 2.2. Expression Analysis

Total RNA was isolated from the cells or tissues using TRIzol reagent (Invitrogen, Carlsbad, CA, USA) and an RNeasy Lipid Tissue Mini Kit (Qiagen, Hilden, Germany). Total RNAs were used to synthesize the cDNA using a ReverTra Ace^®^qPCR RT Master Mix (TOYOBO, Osaka, Japan) with random primers. The cDNA was used to amplify selected genes with THUNDERBRID^®^ SYBR^®^ qPCR Mix (TOYOBO) and primers using the Applied Biosystems QuantStudio 3 Real-Time PCR (Applied Biosystems, Foster city, CA, USA). Expression levels and dene specific primer sets were described previously [28,29,30]. 

For the microarray analysis, total RNAs from C3H10T1/2 adipocytes treated with 10 μM of sesamol or tBHQ for 24 h were isolated and cleaned using an RNeasy Mini Kit (Qiagen, Venlo, The Netherlands). To assess the reproducibility of the RNA amplification, three samples each of DMSO, tBHQ, and sesamol RNA were amplified independently. The cDNA preparation and hybridization to Affymetrix Mouse Genome Arrays of 430 version 2.0 were performed by Macrogen (Seoul, Korea). Data were analyzed using the GeneSpring GX 7.3 software (Agilent Technologies, Santa Clara, CA, USA). 

### 2.3. Luciferase Assay 

The 6XARE-containing luciferase vector was previously described [31]. The ARE-luciferase vector, pcDNA3.1-Nrf2, or pcDNA3.1 empty vector were co-transfected into 293T cells using Lipofectamine 2000 (Invitrogen). For the luciferase reporter assay, the ARE-luciferase vector (200 ng), pcDNA3.1-Nrf2 expressing vector (100 ng), or empty vector, and Lipofectamine 2000 were used for transfection in each well of a 24-well plate. After 48 h of transfection, cells were harvested and the reporter gene activity was measured using the Dual-luciferase Reporter Assay System (Promega, Madison, WI, USA). Sesamol or tBHQ were added to the wells and incubated for an additional 24 h. Luciferase activity was normalized by the Renilla luciferase activity.

### 2.4. Animal Studies.

Male C57BL/6N mice (7 weeks old) obtained from Japan SLC, Inc (Hamamatsu, Shizuoka, Japan) were individually housed in a temperature-controlled room and a 12 h light/dark cycle. After 1 week of adaptation, the mice were randomly divided into four groups: normal diet (ND, 10% fat w/w), HFD (60% fat w/w) orally administrated with vehicle (0.1% DMSO in PBS), control (Ctrl), and HFD treated with two doses of oral administration of sesamol (100 mg/kg/day or 200 mg/kg/day) for 12 weeks. Body weight and food intake were measured twice per week. For the glucose tolerance test, the mice treated for 10 weeks were fasted for 16 h, and blood glucose was determined from tail vein blood at 0, 15, 30, 60, 90, and 120 min after the i.p. (intraperitoneal injection) glucose injection (2 g/kg). The blood glucose level was measured by Blood Glucose Monitor Nocoding 1 Plus (GM01BAA. DAEIL PHARM Co., Ltd., Sungnam, Korea). For the insulin tolerance test, the mice treated for 11 weeks were injected i.p. with insulin (Humulin R, Eli Lilly, Indianapolis, IN, USA) (0.35 U/kg). All animal studies were carried out in accordance with the guidelines of the Animal Research Committee (SKKUIACUC-2018-04-14-3) of Sungkyunkwan University.

Whole-body energy metabolism was measured using Oxylet systems (Panlab, Barcelona, Spain). Mice treated only with sesamol for 3 weeks with no difference in body weight were used to measure energy expenditure. After these mice were placed in metabolic cages and acclimated for 24 h, their oxygen consumption, carbon dioxide release, and energy expenditure were measured for an additional 24 h. 

### 2.5. Statistical Analysis

Data are presented as mean ± s.e.m. Comparisons between the control and experiment groups were analyzed using two-tailed unpaired Student’s t-tests. Statistical significance was defined as *p* < 0.05.

## 3. Results

### 3.1. Sesamol Induces Ucp1 Expression in Adipocytes.

**Figure 1:**
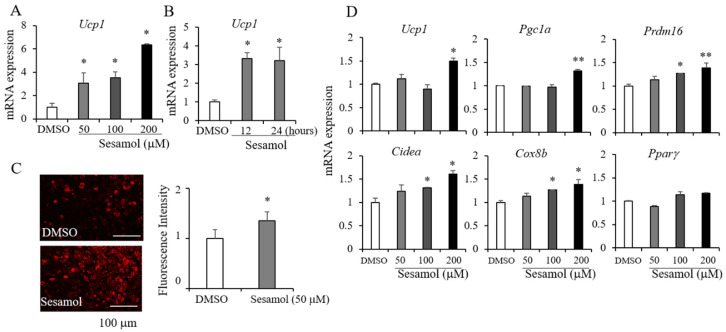
Sesamol induces the expression of *Ucp1* in adipocytes. (**A**) Differentiated C3H10T1/2 adipocytes were treated with sesamol for 12 h at the indicated doses (50, 100, and 200 µM) and the expression of *Ucp1* was measured. (**B**) Primary adipocytes isolated from inguinal adipose tissues were treated with 50 *μ*M of sesamol for 12 or 24 h and the *Ucp1* expression was measured. (**C**) Mitochondrial staining by mitochondria-specific CytoPainter (ab112145) in C3H10T1/2 adipocytes treated with dimethyl sulfoxide (DMSO) or sesamol (50 µM) for 24 h and mitochondrial staining was quantified using the National Institutes of Health’s (NIH) Image J software. (**D**) Differentiated T37i brown adipocytes were treated with sesamol (50, 100, 200 µM) for 12 h and the levels of thermogenic selective markers (*Ucp1*, *Pgc1α*, *Prdm16*, *Cidea*, and *Cox8b*) and *Pparγ* were measured by real-time PCR. Data represent means ± s.e.m. and are representative of three independent experiments. Statistical significance was determined relative to a control using the Student’s *t*-test (* *p* < 0.05; ** *p* < 0.005).

Sesamol, a major lignan found in sesame seeds, has been shown to exhibit anticancer, anti-inflammation, anti-oxidation, and anti-obesity activities [21]. The MTT assay was performed to confirm the non-toxic doses of sesamol. The treatment of C3H10T1/2 cells with sesamol for 24 h did not significantly affect the cell viability (Figure S1). Non-toxic doses of sesamol (50–200 μM) increased the *Ucp1* expression in C3H10T1/2 adipocytes (Figure 1A). In addition, treatments of sesamol at 100 μM for 12 or 24 h induced the *Ucp1* expression in primary adipocytes freshly isolated from inguinal adipose tissues (Figure 1B). Consistently, sesamol increased the mitochondrial contents in C3H10T1/2 adipocytes (Figure 1C). To further investigate the effects of sesamol in brown adipocytes, we treated sesamol into T37i brown adipocytes. Sesamol increased *Ucp1* and other brown adipocyte markers in T37i brown adipocytes but did not affect the expression of the pan adipocyte marker *Ppar*γ** (Figure 1D). Interestingly, other major lignans present in sesame oil did not increase the *Ucp1* expression (Figure S2), which suggested that sesamol plays a selective role in promoting thermogenic adipocytes.

### 3.2. Sesamol Prevents Weight Gains and Metabolic Dysregulation in HFD Fed Obese Mice

**Figure 2:**
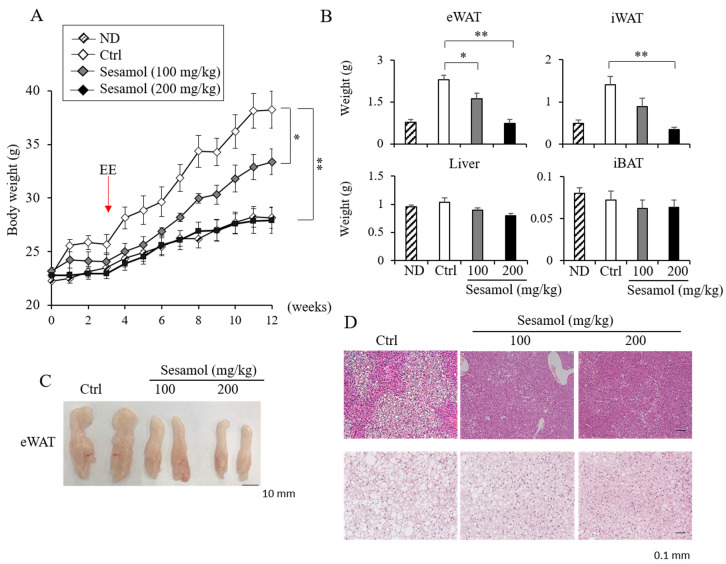
Oral administration of sesamol prevents obesity in high-fat diet (HFD)-induced obese mice. (**A**) Body weights of vehicle control- or sesamol-treated mice. Eight-week-old male C57BL/6J mice were fed with normal diets (ND) or high-fat diets (HFD, 60% fat) and orally administrated with the vehicle control or sesamol (100 mg/kg and 200 mg/kg per day) up to 12 weeks (*n* = 5 per group). Energy expenditure (EE) was measured at 3 weeks of treatments. (**B**) Differences in epididymal white adipose tissue (eWAT), inguinal white adipose tissue (iWAT), liver, and interscapular brown adipose tissue (iBAT) weights in control- and sesamol-treated groups. (**C**) Representative images of eWAT from control- or sesamol-treated mice. Scale bar, 10 mm. (**D**) Representative hematoxylin and eosin (H and E) staining for sections of liver and eWAT from HFD-fed mice. Scale bar, 0.1 mm. Data represent mean ± s.e.m. Statistically significant differences between the control- and sesamol-treated mice were determined by Student’s *t*-test (* *p* < 0.05; ** *p* < 0.005).

**Figure 3:**
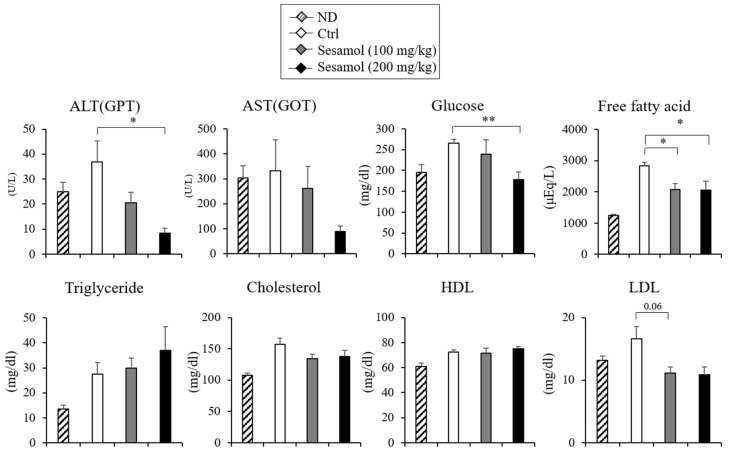
Effects of orally administrated sesamol on serum alanine aminotransferase (ALT; glutamic pyruvic transaminase, GPT), aspartate transaminase (AST; glutamic oxaloacetic transaminase, GOT), glucose and lipid profiles in normal diet (ND)- or high-fat diet (HFD)-fed obese mice. Plasma concentrations of alanine aminotransferase (ALT), aspartate transaminase (AST), glucose, free fatty acids, triglyceride, cholesterol, high-density lipoprotein (HDL), and low-density lipoprotein (LDL) in C57BL/6 mice fed with HFD and administered a daily dose of sesamol at 100 mg/kg or 200 mg/kg (*n* = 5 per group). Data represent means ± s.e.m. and statistically significant differences between the control- and sesamol-treated mice were determined by Student’s t-test (* *p* < 0.05; ** *p* < 0.005).

Given the effect of sesamol in the *Ucp1* induction, we investigated the anti-obese effect of sesamol in HFD-induced obese mice. We administrated sesamol (100 or 200 mg/kg per day) into mice fed with the HFD for 12 weeks. Sesamol compared with the vehicle control mice prevented body weight gain in HFD-fed mice (Figure 2A). The adipose depots’ inguinal white adipose tissue (iWAT) and epididymal white adipose tissue (eWAT) but not the liver and interscapular brown adipose tissue (iBAT) of the sesamol-treated HFD mice weighed less than those of control mice (Figure 2B,C). Food intake in these mice was not different (Figure S3). The histological observation and lipid analysis further revealed a reduced triglyceride accumulation in the liver and iBAT in sesamol-treated HFD mice (Figure 2D). Sesamol also decreased the levels of fasting serum cholesterol and fatty acids levels (Figure 3). The serum alanine aminotransferase (ALT) levels were decreased and the aspartate transaminase (AST) levels were not different (Figure 3), suggesting that the sesamol treatments in the current studies did not exhibit liver toxicity. These data show that sesamol can be used to prevent obesity and its associated metabolic diseases.

### 3.3. Sesamol Increases the Expression of Thermogenic Genes in iWAT and Stimulates Energy Expenditure

**Figure 4:**
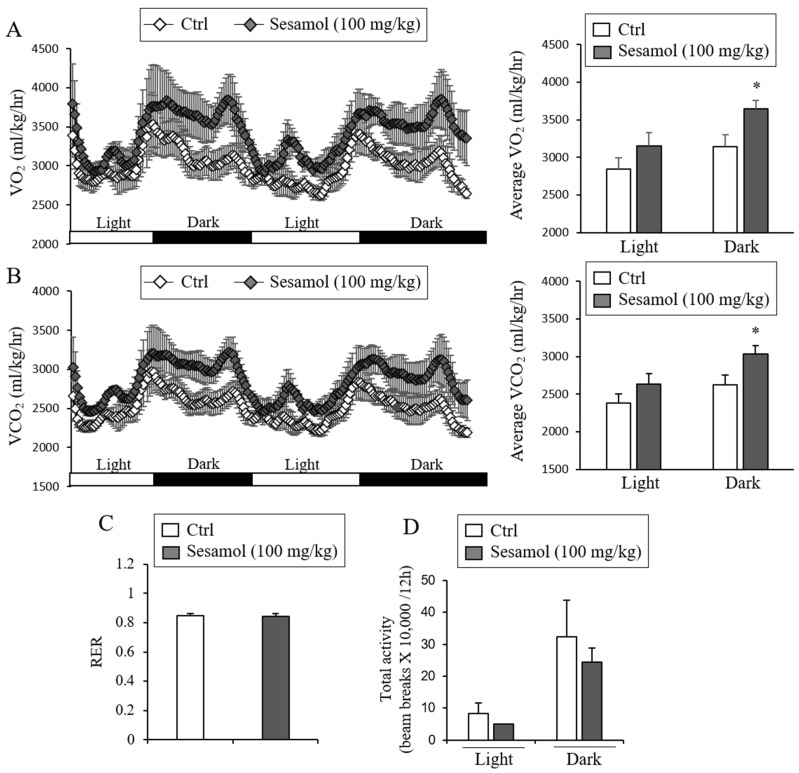
Oral administration of sesamol increases energy expenditure in mice. (**A**, **B**) Energy expenditure was evaluated by measurement of oxygen consumption and carbon dioxide production. O_2_ consumption (**A**) and CO_2_ production rates (**B**) of control- and sesamol-treated mice (100 mg/kg) were measured by indirect calorimetry using CLAMS after 3 weeks on the HFD (*n* = 5 per group). Energy expenditure was measured at 3 weeks of treatments when body weights did not start to diverge. Bar graph (right panel) represents the average of O_2_ consumption or CO_2_ production in each group. (**C**) Respiratory exchange ratio (RER) of the control- and sesamol-treated mice (*n* = 5 per group). (**D**) Total physical activities of control- and sesamol-treated mice (*n* = 5 per group). Data represent mean ± s.e.m. and statistically significant differences between the control- and sesamol-treated mice were determined by Student’s *t*-test (* *p* < 0.05).

**Figure 5:**
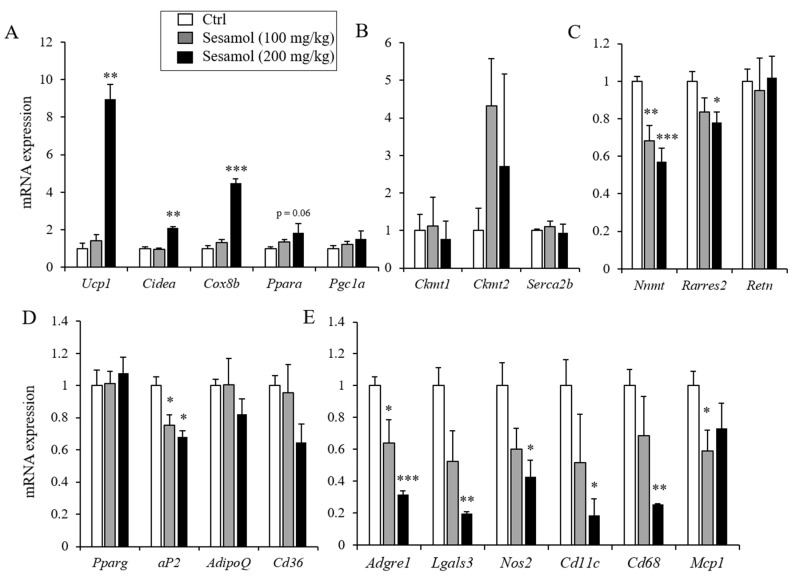
Oral administration of sesamol selectively increases thermogenic markers in white adipose tissue. Gene expression was measured from inguinal white adipose tissue (iWAT) isolated from mice fed with a high-fat diet and orally treated with the vehicle control or sesamol (100 mg/kg and 200 mg/kg per day) (*n* = 5 per group). (**A**) mRNA expression of the thermogenic adipocyte selective genes (*Ucp1*, *Cidea, Cox8b, Ppara* and *Pgc1a*), (**B**) Ucp1-independent thermogenic genes (*Ckmt1*, *Ckmt2*, and *Serca2b*), (**C**) white adipocyte selective genes (*Nnmt, Rarres2,* and *Retn*), (**D**) pan-adipocyte genes (*Pparg*, *aP2*, *AdipoQ*, *Cd36*), and (**E**) inflammatory markers (*Adgre1*, *Lgals3*, *Nos2*, *Cd11c*, *Cd68*, and *Mcp1*) were measured in inguinal adipose tissue by real-time PCR. Data represent means ± s.e.m. Statistically significant differences in the gene expression between the control- (*n* = 5) and sesamol-treated mice (*n* = 5) were determined by Student’s *t*-test (* *p* < 0.05; ** *p* < 0.005; *** *p* < 0.0005).

Since the Ucp1 induction in adipocytes can enhance energy expenditure and prevent obesity and metabolic diseases in diet-induced obese mice [11], we investigated the effect of sesamol on energy expenditure. The treatments of 100 mg/kg of sesamol for up to 6 weeks did not affect the body weight gain in the control- and sesamol-treated groups (Figure 2A). To assess the effects of sesamol in energy expenditure before the significant weight differences were observed, we compared energy expenditure from mice treated with sesamol (100 mg/kg/day) or the vehicle for three weeks. The metabolic analysis showed an increased O_2_ consumption and CO_2_ production in the sesamol-treated group compared with the vehicle control mice (Figure 4A,B). However, the food intake, respiratory exchange ratio (RER), and physical activity were similar between the control- and sesamol-treated groups (Figure 4C,D). Consistent with the increased energy expenditure, thermogenic genes such as *Ucp1*, *Prdm16*, *Cox8b*, and *Cidea*, and the Ucp1 protein were also induced by sesamol, whereas the expression of pan-adipocyte and white adipocyte selective genes was suppressed or not changed by sesamol in iWAT (Figure 5A–D, Figure S4 and Figure S5). The Ucp1-independent thermogenic genes, including creatine kinase mitochondrial 1 (*Ckmt1*), creatine kinase mitochondrial 2 (*Ckmt2*), and sarco/endoplasmic reticulum Ca^2+^-ATPase pump type 2b (*Serca2b*), were not different (Figure 5B). We found that the sesamol treatment down-regulated the expression of pro-inflammatory genes such as adhesion G-protein coupled receptor1 (*Adgrel*), galectin-3 (*Lgals-3*), nitric oxide synthase 2 (*Nos2*), integrin, alpha X (*Cd11c*), cluster of differentiation 68 (*Cd68*), and monocyte chemoattractant protein-1 (*Mcp1*) (Figure 5E). Consistently, we also found improved glucose clearance rates and insulin sensitivity relative to the control mice (Figure S6). These data show that increased energy expenditure by sesamol could be the primary contributor for preventing obesity in the diet-induced obese mice. 

### 3.4. Sesamol Mimics Nrf2 Activation in Adipocytes

**Figure 6:**
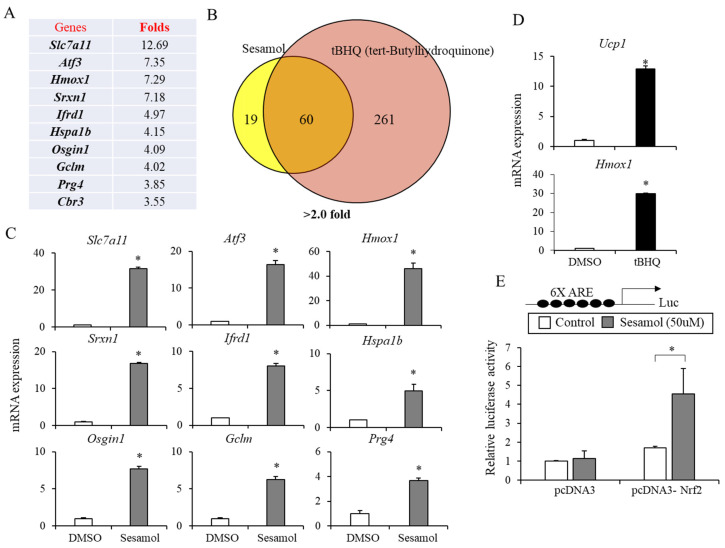
Sesamol stimulates the Nrf2 activation in adipocytes. (**A**) C3H10T1/2 adipocytes were treated with sesamol (50 μM) for 12 h and the gene expression profiles were analyzed by a microarray analysis. Lists of 10 most highly induced genes (solute carrier family 7 member 11*(Slc7a11),* activating transcription factor 3 (*Atf3*), *Hmox1*, *Srxn1*, interferon-related developmental regulator 1 (*Ifrd1*), heat shock protein family a member 1b (*Hspa1b*), oxidative stress induced growth inhibitor 1 (*Osgin1*), *Gclm*, proteoglycan 4 (*Prg4*), and carbonyl reductase 3 (*Cbr3*))by sesamol were shown. (**B**) Expression profiles of C3H10T1/2 adipocytes treated with either sesamol (50 μM) or tert-butylhydroquinone (tBHQ, 50 μM) were compared. Diagram showing the number of genes (>2.0 fold) regulated by sesamol or tBHQ. (**C**) Expression of sesamol (50 μM)-induced genes in C3H10T1/2 adipocytes were confirmed by real-time PCR. (**D**) Induction of the *Ucp1* and *Hmox1* mRNA expressions by tBHQ in C3H10T1/2 adipocytes. (**E**) HEK293T cells were transfected with the Nrf2-dependent antioxidant response element (ARE) luciferase reporter construct (6XARE) and an expression vector coding for Nrf2 (pcDNA3-Nrf2). After 36 h, cells were treated with DMSO (control) or sesamol for an additional 12 h and the firefly luciferase activity was measured. The firefly luciferase enzyme activity was normalized to the Renilla luciferase enzyme activity. Data are means ± standard deviations (*n* = 3). Statistical significance was determined by comparison with the control using Student’s *t*-test (* *p* < 0.05).

Having found that sesamol increases energy expenditure, we sought to determine its molecular mechanisms in adipocytes by performing a microarray analysis in C3H10T/12 adipocytes. Seventy-nine genes were induced at least two-fold or higher. Interestingly, 7 of 10 genes most highly induced by sesamol (>3.55-fold), namely *Slc7a11*, *Atf3*, *Hmox1*, *Sxrn1*, *Osgin1*, *Gclm*, and *Cbr3,* have been previously known as Nrf2 target genes (Figure 6A) [20]. Thus, we compared sesamol and known Nrf2 activators, tBHQ-induced genes. Seventy-six percent of genes (60 out of 79) induced by sesamol were also overlapped with those by tBHQ in the microarray analysis (Figure 6B). We confirmed that sesamol increased the expression of most highly induced genes *Slc7a11, Atf3, Hmox1*, *Srxn1*, *Ifrd1*, *Hspa1b*, *Osgin1*, *Glcm*, and *Prg4* by real-time PCR in C3H10T1/2 adipocytes (Figure 6C). A similar induction of *Hmox1* and *Atf3* by sesamol in T37i brown adipocytes further corroborated the sesamol-mediated Nrf2 activation (Figure S7). Consistently, the tBHQ treatments also induced the expression of the Nrf2 target genes *Hmox1* and *Ucp1* in C3H10T1/2 and primary adipocytes (Figure 6D and Figure S8). These data suggested the possibility of the Nrf2 activation as a functional mediator of the thermogenic responses to sesamol. 

The transcriptional activation of ARE-containing genes by Nrf2 plays a critical role in the cellular defense system [19,20]. To further show the effects of sesamol in the Nrf2 activation, we transiently expressed Nrf2 and the luciferase reporter constructs of the ARE promoter. The luciferase reporter constructs carrying the 6X repeated ARE 5′- region were transiently transfected into HEK293T cells and the relative luciferase activity was assessed. The expression of Nrf2 with the ARE promoter luciferase constructs showed a modest increase in the luciferase activity. This effect was significantly increased (up to three-fold) by the sesamol treatment (Figure 6E). These data suggest that sesamol can directly stimulate the Nrf2-mediated ARE promoter transcription. 

### 3.5. Nrf2 is Essential for The Sesamol-Mediated Ucp1 Induction

**Figure 7:**
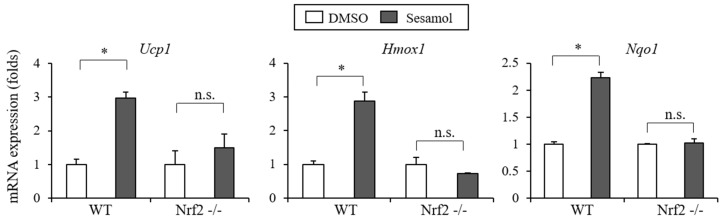
Nrf2 is essential for the sesamol-mediated Ucp1 induction. Induction of *Ucp1* and Nrf2 target genes by sesamol was compromised in the absence of the Nrf2 expression. Nrf2 knockout mouse embryonic fibroblasts (Nrf2 KO MEF) or Nrf2 wild type cells (Nrf2 WT) were treated with sesamol for 12 h and relative levels of *Ucp1*, *Hmox1*, and *Nqo1* were measured by real-time PCR. Data are means ± standard deviations (*n* = 3). Data represent means ± s.e.m. and are representative of three independent experiments. Statistical significance was determined relative to a control using the Student’s *t*-test (ns, not significant; * *p* < 0.05). WT, Nrf2 wild type cells; Nrf2 -/-, Nrf2 KO MEF.

Having observed the biological actions of sesamol as an Nrf2 activator in adipocytes, we reasoned that Nrf2 plays an essential role in sesamol’s effects in adipocytes. To show the necessity of Nrf2 in sesamol’s influence, we treated wild type cells (Nrf2 WT) and Nrf2 knockout (KO) MEFs with sesamol. As expected, sesamol effectively induced *Ucp1* and the Nrf2 target genes *Hmox1* and *Nqo1* in the Nrf2 WT cells, but the sesamol-regulated expressions of *Ucp1, Hmox1,* and *Nqo1* were significantly compromised in the Nrf2 KO MEFs (Figure 7), indicating the necessity of Nrf2 in the sesamol-mediated effects. These results further suggest that the sesamol-mediated Nrf2 activation plays an essential role in the regulation of the *Ucp1* induction in adipocytes. 

## 4. Discussion

The stimulation of brown-like adipocytes protects mice against the development of diet-induced obesity and metabolic diseases [32,33,34]. Similarly, an increased BAT activity in humans upon cold exposure enhances energy expenditure [35,36,37]. These observations prompted researchers to attempt to identify the stimulators that induce thermogenic adipocytes. The thermogenic stimulators include the phytochemicals berberine, butein, capsaicin, dihydroxyflavone, and fucoxanthin [11]. In this study, we showed that sesamol induces the *Ucp1* expression in white adipocytes and increases energy expenditure in mice. The stimulation of energy expenditure seems to be a causative factor for preventing obesity and metabolic diseases in high-energy food-fed obese mice. We measured the energy expenditure in control and sesamol orally administrated mice for 3 weeks, when the body weights were not different between these groups. We observed an increased O_2_ consumption and CO_2_ production in the HFD-fed mice treated compared with the vehicle control HFD-fed mice. Thus, an increased energy expenditure can directly contribute to the observed effects in preventing weight gain and in improving glucose metabolism in mice receiving high-fat diets. Therefore, the stimulation of thermogenic adipocytes by sesamol might be a valuable strategy for developing new therapeutic interventions for obesity and its associated metabolic diseases.

Our findings are consistent with previous studies showing the effects of sesamol in obesity and glucose metabolism [23,24,25]. However, its underlying mechanism has not been clearly elucidated. Here, we showed that sesamol increased the expression of *Ucp1* and antioxidant genes in white adipocytes via the Nrf2 activation, leading to increasing the energy expenditure and ameliorating the metabolic dysregulation. Given the molecular mechanisms involved in the Nrf2 activation by sesamol in adipocytes, we can speculate that other Nrf2 activating phytochemicals may also increase the *Ucp1* expression in white adipocytes and enhance energy expenditure. In line with this, the Nrf2 activator sulforaphane, which is found in broccoli, and its analog glucoraphanin improve glucose control in humans and prevent obesity in mice [18,38]. It has also been proposed that enhanced Nrf2 signaling by the Keap1 knockdown prevents hepatic steatosis, dyslipidemia, and insulin resistance [39]. Similarly, the Keap1-hypo allele (Nrf2 activation) mice were partially protected from obesity, had lower fasting glucose and insulin levels, and developed less liver steatosis [40]. In addition, sesamol has been shown to protect cognitive impairments by activating the Nrf2 transcriptional pathway [41]. Therefore, the identification of a potent Nrf2 activator may provide another tool to prevent obesity and its associated metabolic diseases by enhancing energy expenditure. 

Our data have demonstrated the role of the sesamol-mediated Nrf2 activation in regulating the *Ucp1* expression and energy expenditure in white adipose tissues. Similarly, prior studies have shown that the Nrf2 modulation can affect energy expenditure and prevent weight gain. Nrf2 polymorphisms are associated with diabetes and obesity in humans [42]. However, Nrf2 is the focal point that incorporates various signaling pathways, including cell stress and cell proliferation. Unlike the potential benefits of anti-oxidation by the Nrf2 activation, the Nrf2 deletion can also increase the energy expenditure, Ucp1 expression, and prevent metabolic dysfunction [43]. Nrf2 deficiency in Lep(ob/ob) mice also reduced the WAT mass and prevented a hepatic lipid accumulation, but induced insulin resistance and dyslipidemia [44]. Thus, the developmental Nrf2 signaling complicates the actions in energy expenditure and obesity because a genetic deficiency is associated with a reduced fat mass. Furthermore, Nrf2 is a ubiquitous and pleiotropic factor in various metabolic tissues. Thus, the potential adverse and tissue-specific effects of Nrf2 should be further assessed in the future. Toward this end, the adipose-selective activation of Nrf2 in adult mice may limit the unwanted side-effects and improve the clinical outcomes for the treatment of obesity and glucose metabolism by increasing the energy expenditure. 

Recent studies in adipocytes have indicated the existence of functional Ucp1-dependent and Ucp1-independent thermogenic regulators [14]. Sesamol can also work by increasing thermogenesis in a Ucp1-independent manner. Consistently, a limitation of the current study is in the failure to determine the thermogenic effects of sesamol in a Ucp1-dependenent manner. The administration of sesamol in Ucp1 KO mice can address this Ucp1 dependency. Additionally, several browning agents can induce stress such as heat loss, which leads to an increased WAT browning and energy expenditure [45]. In this case, WAT browning can be a secondary consequence of the enhanced heat loss associated with the changes in insulation or sympathetic events. Our current studies cannot exclude the possibility of sesamol as a secondary browning agent. Future experiments on the administration of sesamol in mice at thermoneutrality would resolve this limitation.

Our data suggest that dietary sesamol could be beneficial in increasing energy expenditure and preventing metabolic diseases in humans. Since there are differences in the BAT activities, the Ucp1 expression levels in WAT, and the living conditions between human and mice, the effect of sesamol on the Ucp1 induction in humans could be conflicting [14]. Thus, the possible effects of sesamol supplementation in the human physiology should be investigated in the future.

## 5. Conclusions

In conclusion, our data have revealed the potential benefits of sesamol in diet-induced obese mice. The mechanism studies indicated that the Nrf2 activation by sesamol induces Ucp1 and energy expenditure, leading to the prevention of weight gain and metabolic diseases. These findings suggest that the phytochemical sesamol and its target, the Nrf2 activation, in adipocytes might be useful tools for developing treatments for obesity and related metabolic diseases. 

## Supplementary Materials

The following are available online at https://www.mdpi.com/2072-6643/12/5/1459/s1, Figure S1: Effects of sesamol on cell viability, Figure S2: Induction of the Ucp1 expression in adipocytes by sesame lignans, Figure S3: Daily food intake in control- or sesamol-treated HFD-fed obese mice, Figure S4: Induction of the Ucp1 protein expression by sesamol in HFD-induced obese mice, Figure S5: Uncropped versions of immunoblots in the figure S4. Figure S6: Sesamol improves glucose and insulin tolerance in obese mice, Figure S7: Sesamol induces the expression of Nrf2 target genes in T37i brown adipocytes, Figure S8: tBHQ induces the expression of thermogenic genes in primary adipocytes.
