# Supplementary material for: Sesamol Increases Ucp1 Expression in White Adipose Tissues and Stimulates Energy Expenditure in High-Fat Diet-Fed Obese Mice
Source: Nutrients. 2020 May 18;12(5):1459. doi: 10.3390/nu12051459 (PMC7284577; doi:10.3390/nu12051459)
Supplement: Supplementary file 1 [file nutrients-12-01459-s001.pdf]

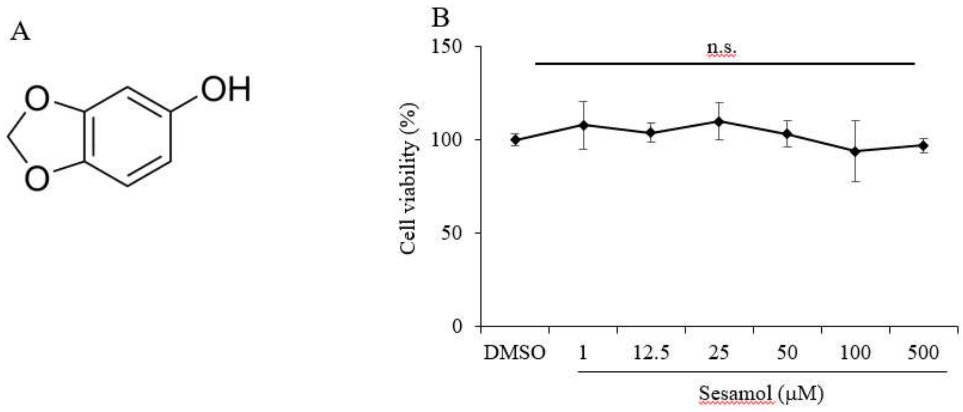

Figure S1. Cell viability of C3H10T1/2 cells with sesamol treatment at the indicated concentrations. (A) Chemical structure of sesamol. (B) Cells were treated with dimethylsulfoxide (DMSO, ctrl) or sesamol at the indicated doses for 24 hour, and cell viability was determined by MTT assays. Data are means ± SEM from three independent experiments.

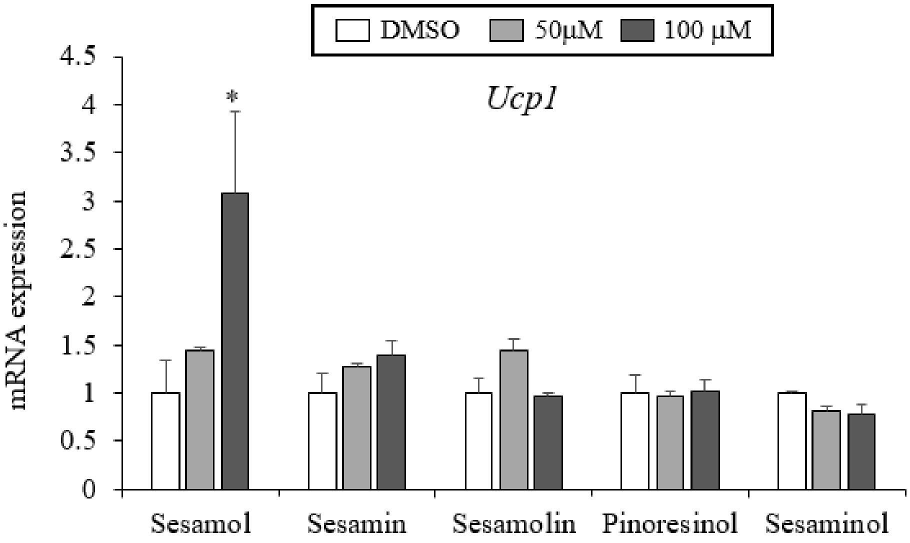

Figure S2. Differentiated C3H10T1/2 adipocytes were treated with major lignans present in sesame oil for 12 hours and the expression of *Ucp1 was* measured. Data represent means ± s.e.m. and are representative of three independent experiments. Statistical significance was determined relative to a control using the Student's *t*-test (* $p < 0.05$).

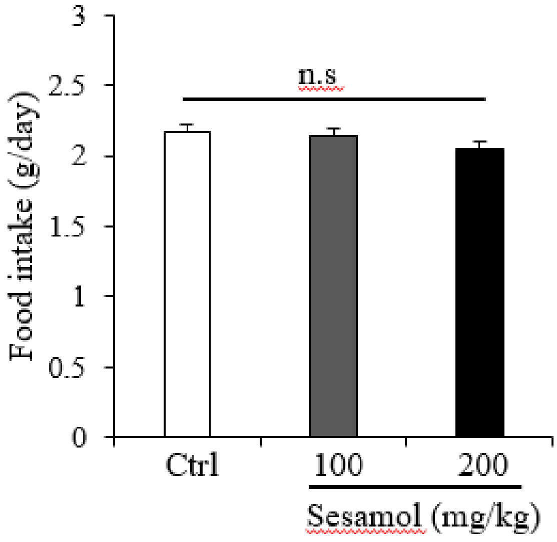

Figure S3. Daily food intake was measured twice per week for 12 weeks. Averages of daily food intake of control or sesamol treated groups (n=5 per group). Data represent means ± s.e.m. Food intake among groups is not considered significant (ns, not significant).

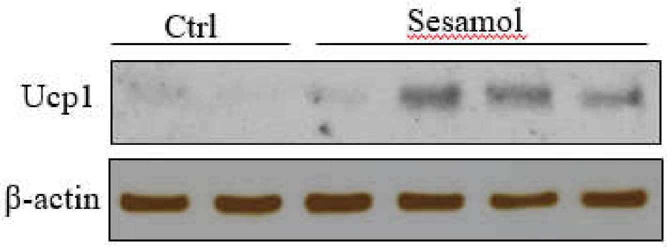

Figure S4. Expression of Ucp1 and β-actin protein in iWAT of control and sesamol treated mice. Expression levels were determined by western blotting. Eight-week-old male C57BL/6J mice were fed with a high fat diet (HFD, 60% fat) and orally administrated with vehicle control or sesamol (200 mg/kg per day) upto 12 weeks.

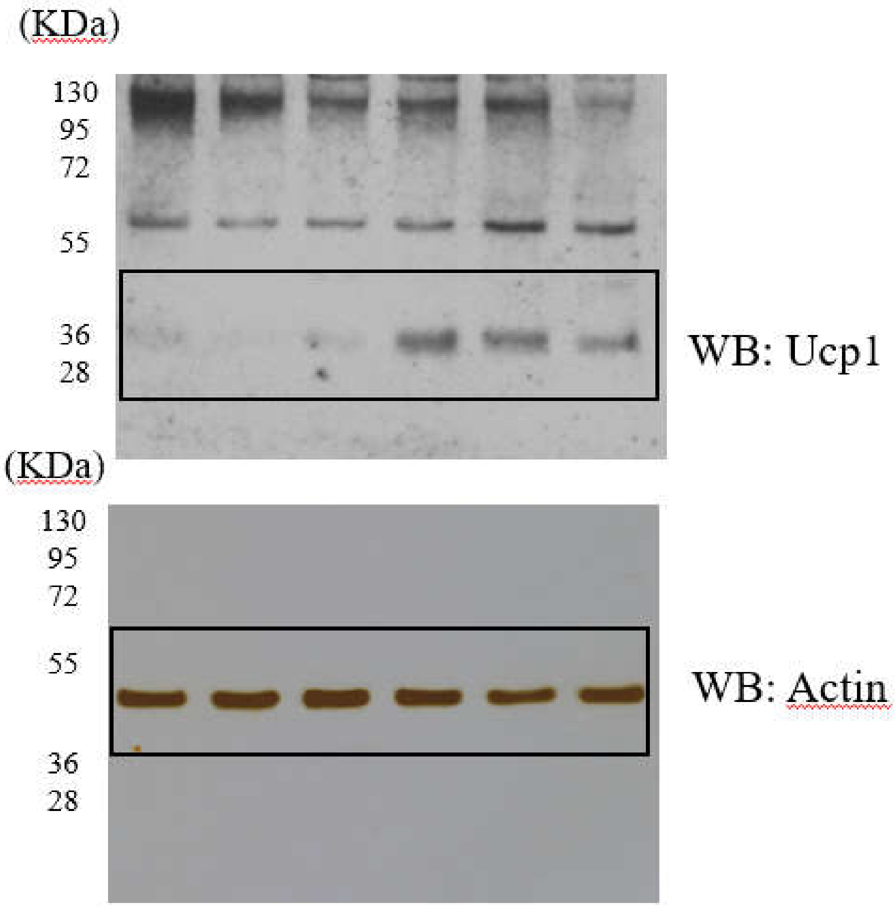

Figure S5. Uncropped versions of immunoblots in the figure S4.

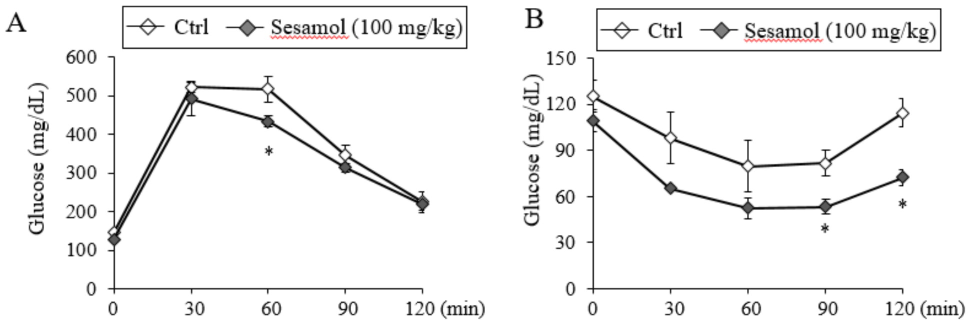

Figure S6. (A) Glucose tolerance test and (B) Insulin tolerance test in control and sesamol-treated groups (n=5 per group). Mice were fasted for 6 hours before the intraperitoneal injection of glucose or insulin for Glucose Tolerance Test (GTT) and Insulin Tolerance Test (ITT). Tail blood samples were collected at different time points for measurement of blood glucose. Data represent means ± s.e.m. and statistically significant differences in the control and sesamol-treated mice were determined by Student's *t*-test (* $P < 0.05$).

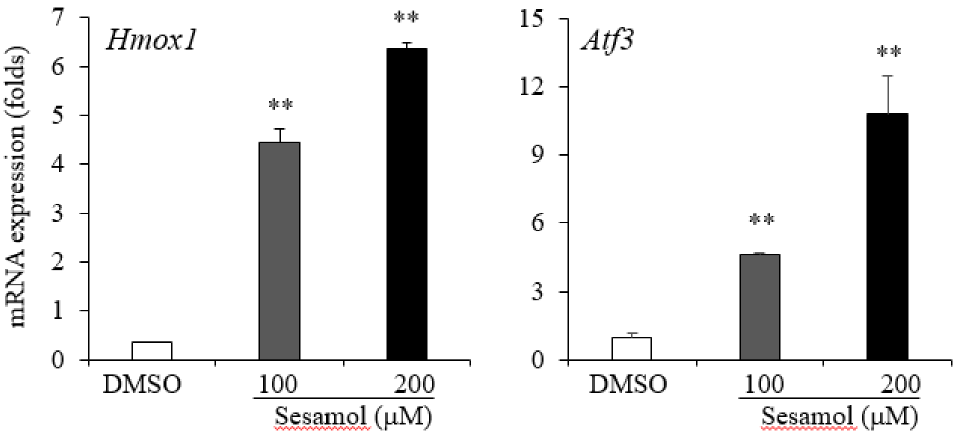

Figure S7. Differentiated T37i brown adipocytes were treated with sesamol (100 or 200 μM) for 12 hours and the levels of Nrf2 target genes (*Hmox1* and *Atf3*) were measured by real-time PCR. Data represent means ± s.e.m. and are representative of three independent experiments. Statistical significance was determined relative to a control using the Student's *t*-test (** $p < 0.005$).

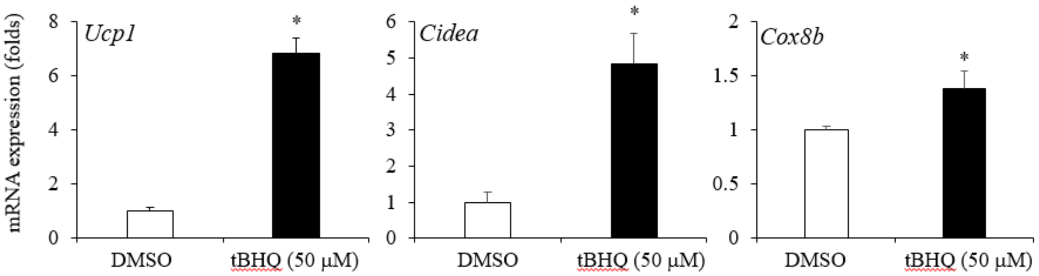

Figure S8. Primary adipocytes freshly isolated from inguinal adipose tissues were treated with tBHQ (50 μM) for 24 hours and the levels of thermogenic genes were measured by real-time PCR. Data represent means ± s.e.m. and are representative of three independent experiments. Statistical significance was determined relative to a control using the Student's *t*-test (* $p < 0.05$).
